# Therapeutic Potential of Cannabinoid Receptors Type 1 and 2—Novel Insights for Enhancing the Chance of Clinical Success

**DOI:** 10.3390/ph18091324

**Published:** 2025-09-04

**Authors:** Uwe Grether

**Affiliations:** Roche Pharma Research and Early Development, Roche Innovation Center Basel, F. Hoffmann-La Roche Ltd., Grenzacherstrasse 124, 4070 Basel, Switzerland; uwe.grether@roche.com

This Special Issue of *Pharmaceuticals* presents eight original articles and three reviews, underscoring the ongoing robust interest in research on cannabinoid receptor type 1 (CB_1_R) and type 2 (CB_2_R) more than 30 years after their discovery [1,2].

The endocannabinoid system (ECS) is a critical lipid signaling network present in all vertebrates, playing a central role in maintaining body homeostasis. It regulates a range in physiological and pathological processes, including chronic pain, multiple sclerosis, Alzheimer’s disease, obesity, diabetes, and kidney disorders, all of which contribute significantly to global health and socioeconomic burdens [3,4,5]. The ECS comprises lipid signaling molecules known as endocannabinoids (eCBs), their receptors (primarily CB_1_R and CB_2_R), biosynthetic and hydrolytic enzymes, and transporters.

CB_1_R, the most abundant G protein-coupled receptor (GPCR) in the brain, and CB_2_R, widely distributed on immune cells, are the primary molecular targets within the ECS [6]. These receptors regulate critical functions such as sleep, body temperature, energy balance, memory, emotional processing, and immune response [7]. The best-studied eCBs are anandamide (AEA) and 2-arachidonoylglycerol (2-AG), which act as endogenous agonists for CB_1_R and CB_2_R [8]. Dysregulation of these receptors has been linked to metabolic disorders (e.g., obesity, diabetes), cardiovascular diseases (e.g., heart failure, kidney failure), and neurological disorders (e.g., epilepsy, Alzheimer’s, Parkinson’s, and multiple sclerosis) [9,10].

Plant-derived cannabinoids, such as (–)-Δ9-trans-tetrahydrocannabinol (THC) and cannabidiol (CBD), have garnered significant attention for their therapeutic potential. Beyond the psychoactive effects of THC, cannabis plants contain over 140 phytocannabinoids, many of which lack psychoactive properties but exhibit health-promoting effects [11]. For instance, CBD has shown efficacy in treating drug-resistant epilepsy (e.g., Dravet and Lennox–Gastaut syndrome) [12] and is being explored for conditions like hyperphagia, obesity, and Prader–Willi syndrome [13]. However, the unregulated promotion of cannabis-based products without sufficient evidence of efficacy, safety, or quality control poses risks to consumers [14,15].

The pharmaceutical industry has made substantial progress in developing CB_1_R and CB_2_R modulators, with over 5000 patents filed since 1970 and more than 150 clinical trials underway. Recent advancements, such as the generation of CB_1_R and CB_2_R 3D structures [16], the discovery of allosteric binding sites [17], and insights into biased signaling mechanisms [18,19], offer promising avenues for designing next-generation drugs. These developments could address the current limitations of clinical success and unlock the full therapeutic potential of these receptors.

Despite these advances, challenges persist. Not all diseases benefit equally from CB_1_R/CB_2_R modulation, and gaps in preclinical and clinical knowledge hinder the translation of basic research into effective therapies [20,21]. Collaborative efforts are needed to standardize protocols, share reproducible data, and reach a consensus on the most suitable indications for CB_1_R/CB_2_R-targeted drugs. Such efforts could accelerate the development of personalized medicine and evidence-based cannabinoid therapies, particularly for orphan diseases.

The ECS represents a promising frontier for therapeutic innovation, with CB_1_R and CB_2_R playing pivotal roles in addressing a wide array of central and peripheral diseases. Recent advancements, such as novel ligand development, the discovery of allosteric binding sites, and insights into biased signaling, have opened new opportunities for designing next-generation drugs. Translating validated preclinical and clinical research into industrial, clinical, and educational frameworks could expedite the development of safe and effective cannabinoid-based treatments, ultimately improving patient outcomes and public understanding of this complex system.

This Special Issue of *Pharmaceuticals* compiles cutting-edge research to advance CB_1_R- and CB_2_R-targeted therapies. It features studies on novel compounds, mechanistic insights, and translational findings, covering topics from cannabis toxicity and pharmacokinetics to cannabinoids for neurocognitive disorders and drug repurposing. The collection also explores allosteric modulators, fatty acid amide hydrolase (FAAH) inhibition for sleep, and critical drug–cannabinoid interactions. Additionally, it presents research on cisplatin’s impact on the ECS, synergistic cancer treatments, novel approaches for colitis, and the broader role of cannabinoids in sleep and addiction, emphasizing the potential of endocannabinoidome targeting for inflammatory bowel disease.

By addressing knowledge gaps and innovative approaches, the articles in this Special Issue aim to catalyze the advancement of ECS research into tangible therapeutic applications. Together, these contributions highlight the potential of ECS modulation to revolutionize the treatment of human diseases and lay a foundation for further progress in this exciting field.

The study by Filipiuc et al. (contribution 1) investigated the acute toxicity and pharmacokinetics of an EU-GMP-certified *Cannabis sativa* L. strain (15.6% THC, <1% CBD) using female Sprague-Dawley rats. Following OECD 423 guidelines, the oral LD50 exceeded 5000 mg/kg (human equivalent: ~806 mg/kg), with no significant toxicity, mortality, or organ abnormalities observed, though mild sedation and motor incoordination occurred at higher doses. Biochemical and histopathological analyses confirmed no adverse effects on liver or kidney function. Pharmacokinetics revealed elevated plasma levels of acid precursors like tetrahydrocannabinolic acid isomer A (THCA-A) and cannabigerolic acid (CBGA), which enhance therapeutic potential without psychotropic effects. These findings support the safety and potential clinical use of this strain for conditions such as chronic pain and neurodegenerative diseases, emphasizing the importance of a high THCA-A:THC ratio to reduce psychotropic risks while preserving therapeutic benefits.

Williams et al. (contribution 2) explored the therapeutic potential of CBD and its synthetic analog HU308 in mitigating HIV-associated neurocognitive disorders (HANDs). HANDs, driven by chronic inflammation and viral persistence in the CNS, remain prevalent despite combined antiretroviral therapy (cART). The study showed that CBD and HU308 reduced extracellular vesicles (EVs) and viral RNA (TAR and env), along with proinflammatory cytokines (IL-1β, TNF-α). Using in vitro models, 3D neurospheres, and a humanized mouse model, HU308 combined with low-dose cART provided significant reductions in viral replication and inflammation compared to cART alone. These findings suggest that CBD and HU308 may complement cART in addressing HANDs by targeting both viral and inflammatory pathways.

Criscuolo et al. (contribution 3) combined computational screening and experimental validation to identify FDA-approved drugs with CB_1_R activity for drug repurposing. Compounds such as Raltegravir, Methotrexate, and Miltefosine displaced [^3^H]CP55,940 in binding assays without interacting with key endocannabinoid-metabolizing enzymes, indicating minimal off-target effects. Miltefosine demonstrated CB_1_R-mediated anti-proliferative effects in HaCaT cells. These results highlight the potential of drug repurposing to expedite CB_1_R-targeted drug discovery while reducing costs and timelines.

Green et al. (contribution 4) used molecular modeling and mutagenesis to identify key residues for positive allosteric modulators (PAMs) at CB_1_R, a promising target for neuropathic pain and addiction therapies. Six allosteric sites were identified, including novel cholesterol-binding sites. The ZCZ011 binding site (Site 3) was essential for allosteric agonism, as mutations at F191A^3.27^ and I169A^2.56^ abolished G protein dissociation induced by PAMs. These findings underscore the complexity of allosteric modulation and the need for detailed receptor–ligand studies to develop selective CB_1_R modulators.

Martin et al. (contribution 5) examined FAAH inhibition for sleep promotion in TauP301S (PS19) mice, a model of neurodegeneration. Pharmacological FAAH inhibition improved sleep behaviors, but complete genetic FAAH knockout failed to prevent sleep loss, neuroinflammation, or cognitive decline. These results suggest FAAH as a promising sleep-promoting target in neurodegenerative diseases, though dosing and timing require further investigation to avoid potential long-term drawbacks.

Campos et al. (contribution 6) reviewed drug–cannabinoid interactions in therapies for epilepsy, autism, cancer, multiple sclerosis, and pain. Cannabinoids, particularly THC and CBD, influence cytochrome P450 enzymes (e.g., CYP2C9, CYP3A4), altering the plasma concentrations of co-administered drugs. Examples include CBD increasing clobazam levels in epilepsy and interfering with tamoxifen metabolism in cancer. The review emphasizes the need for personalized monitoring of drug interactions to optimize therapeutic efficacy and minimize toxicity.

López-Tofiño et al. (contribution 7) studied cisplatin-induced changes in the ECS of male rats to understand chemotherapy side effects. A single subnoxious dose of cisplatin caused significant ECS alterations, including reduced MAGL expression in the gastrointestinal system and nervous system changes resembling chronic pain conditions. These findings suggest ECS modulation as a potential strategy to mitigate chemotherapy-induced toxicities.

Gong et al. (contribution 8) demonstrated synergistic anti-tumor effects by combining AXL inhibition with the cannabinoid WIN55212-2. The combination reduced tumor growth in vitro and in vivo, enhanced apoptosis, and increased CD8^+^ T-cell infiltration in immunocompetent mice. These results highlight AXL as a key target to sensitize cannabinoids for cancer therapy by modulating both tumor cells and the tumor microenvironment.

Thapa et al. (contribution 9) showed that combining low-dose THC with either ZCZ011 or CBD effectively alleviated DSS-induced colitis in mice. The combinations improved inflammation markers, restored GLP-1 homeostasis, and mitigated metabolic complications without significant toxicity. This synergistic approach offers a safer therapeutic strategy for ulcerative colitis, targeting both inflammatory and metabolic pathways.

Pérez-Morales et al. (contribution 10) reviewed the role of CB_1_R and CB_2_R ligands in sleep disorders and addiction. Cannabinoids generally promote sleep in animal models and may benefit insomnia and sleep apnea in humans, though long-term clinical data is limited. For addiction, CB_1_R antagonists and CB_2_R agonists show promise in reducing drug-seeking behaviors in animal models, though adverse psychiatric effects have limited CB_1_R antagonist use in humans. The review calls for rigorous research to better understand the benefits and risks of therapeutic cannabinoids.

Thapa et al. (contribution 11) reviewed the endocannabinoidome (eCBome) as a therapeutic target for IBD and its extraintestinal manifestations (EIMs). Modulating the eCBome offers potential for managing systemic complications like arthritis and liver dysfunction in addition to intestinal inflammation. The review highlights the need for mechanistic studies to leverage the eCBome for holistic IBD management.

Together, these contributions emphasize the potential of ECS modulation to revolutionize therapeutic strategies, addressing a wide range of human diseases and laying a foundation for future research and clinical applications.

## References

[1] Matsuda L.A., Lolait S.J., Brownstein M.J., Young A.C., Bonner T.I. (1990). Structure of a Cannabinoid Receptor and Functional Expression of the Cloned CDNA. Nature.

[2] Munro S., Thomas K.L., Abu-Shaar M. (1993). Molecular Characterization of a Peripheral Receptor for Cannabinoids. Nature.

[3] Mechoulam R., Parker L.A. (2013). The Endocannabinoid System and the Brain. Annu. Rev. Psychol..

[4] Marzo V.D., Piscitelli F. (2015). The Endocannabinoid System and Its Modulation by Phytocannabinoids. Neurotherapeutics.

[5] Maccarrone M., Marzo V.D., Gertsch J., Grether U., Howlett A.C., Hua T., Makriyannis A., Piomelli D., Ueda N., van der Stelt M. (2023). Goods and Bads of the Endocannabinoid System as a Therapeutic Target: Lessons Learned after 30 Years. Pharmacol. Rev..

[6] Howlett A.C. (2005). Cannabinoid receptor signaling. Handbook of Experimental Pharmacology.

[7] Piomelli D. (2003). The Molecular Logic of Endocannabinoid Signalling. Nat. Rev. Neurosci..

[8] Pertwee R.G. (2015). Endocannabinoids and their pharmacological actions. Handbook of Experimental Pharmacology.

[9] Pacher P., Kunos G. (2013). Modulating the Endocannabinoid System in Human Health and Disease--Successes and Failures. FEBS J..

[10] Zou S., Kumar U. (2018). Cannabinoid Receptors and the Endocannabinoid System: Signaling and Function in the Central Nervous System. Int. J. Mol. Sci..

[11] Radwan M.M., Chandra S., Gul S., ElSohly M.A. (2021). Cannabinoids, Phenolics, Terpenes and Alkaloids of Cannabis. Molecules.

[12] Devinsky O., Marsh E., Friedman D., Thiele E., Laux L., Sullivan J., Miller I., Flamini R., Wilfong A., Filloux F. (2016). Cannabidiol in Patients with Treatment-Resistant Epilepsy: An Open-Label Interventional Trial. Lancet Neurol..

[13] Russo E.B., Marcu J. (2017). Cannabis Pharmacology: The Usual Suspects and a Few Promising Leads. Adv. Pharmacol..

[14] Volkow N.D., Swanson J.M., Evins A.E., DeLisi L.E., Meier M.H., Gonzalez R., Bloomfield M.A.P., Curran H.V., Baler R. (2016). Effects of Cannabis Use on Human Behavior, Including Cognition, Motivation, and Psychosis: A Review. JAMA Psychiatry.

[15] Bonn-Miller M.O., Loflin M.J.E., Thomas B.F., Marcu J.P., Hyke T., Vandrey R. (2017). Labeling Accuracy of Cannabidiol Extracts Sold Online. JAMA.

[16] Li X., Shen L., Hua T., Liu Z.-J. (2020). Structural and Functional Insights into Cannabinoid Receptors. Trends Pharmacol. Sci..

[17] Morales P., Goya P., Jagerovic N., Hernandez-Folgado L. (2016). Allosteric Modulators of the CB(1) Cannabinoid Receptor: A Structural Update Review. Cannabis Cannabinoid Res..

[18] Tian L., Qiang T., Liu S., Zhang B., Zhang Y., Zhang B., Hu J., Zhang J., Lu Q., Ke C. (2025). Cannabinoid Receptor 1 Ligands: Biased Signaling Mechanisms Driving Functionally Selective Drug Discovery. Pharmacol. Ther..

[19] Morales-Pastor A., Miljuš T., Dieguez-Eceolaza M., Stępniewski T.M., Ledesma-Martin V., Heydenreich F.M., Flock T., Plouffe B., Gouill C.L., Duchaine J. (2025). Multiple Intramolecular Triggers Converge to Preferential G Protein Coupling in the CB(2)R. Nat. Commun..

[20] Cristino L., Bisogno T., Marzo V.D. (2020). Cannabinoids and the Expanded Endocannabinoid System in Neurological Disorders. Nat. Rev. Neurol..

[21] Gasperi V., Guzzo T., Topai A., Gambacorta N., Ciriaco F., Nicolotti O., Maccarrone M. (2023). Recent Advances on Type-2 Cannabinoid (CB(2)) Receptor Agonists and Their Therapeutic Potential. Curr. Med. Chem..

